# EMF exposure variation among MRI sequences from pediatric examination protocols

**DOI:** 10.1002/bem.22159

**Published:** 2018-11-30

**Authors:** Jennifer Frankel, Kjell Hansson Mild, Johan Olsrud, Jonna Wilén

**Affiliations:** ^1^ Department of Radiation Sciences Radiation Physics Umeå University Umeå Sweden; ^2^ Center for Medical Imaging and Physiology Skåne University Hospital Lund Sweden

**Keywords:** children, electromagnetic field, epidemiology, exposure assessment, radiofrequency

## Abstract

The magnetic resonance imaging (MRI) exposure environment is unique due to the mixture and intensity of magnetic fields involved. Current safety regulations are based on well‐known acute effects of heating and neuroexcitation while the scientific grounds for possible long‐term effects from MRI exposure are lacking. Epidemiological research requires careful exposure characterization, and as a first step toward improved exposure assessment we set out to characterize the MRI‐patient exposure environment. Seven MRI sequences were run on a 3‐Tesla scanner while the radiofrequency and gradient magnetic fields were measured inside the scanner bore. The sequences were compared in terms of 14 different exposure parameters. To study within–sequence variability, we varied sequence settings such as flip angle and slice thickness one at a time, to determine if they had any impact on exposure endpoints. There were significant differences between two or more sequences for all fourteen exposure parameters. Within–sequence differences were up to 60% of the corresponding between‐sequence differences, and a 5–8 fold exposure increase was caused by variations in flip angle, slice spacing, and field of view. MRI exposure is therefore not only sequence‐specific but also patient‐ and examination occurrence‐specific, a complexity that requires careful consideration for an MRI exposure assessment in epidemiological studies to be meaningful. Bioelectromagnetics. 40:3–15, 2019. © 2018 The Authors. *Bioelectromagnetics* Published by Wiley Periodicals, Inc.

## INTRODUCTION

Magnetic resonance imaging (MRI) is a medical imaging technique used in all areas of medicine, and it has steadily increased in popularity since its introduction more than 30 years ago. An MRI examination consists of different imaging sequences designed to produce detailed internal anatomical images. The generation of MR images requires a strong static magnetic field, a switched gradient field, and a pulsed radiofrequency (RF) field. Today's clinical scanners operate with a static magnetic field of either 1.5 or 3 Tesla. The switched gradient field provides temporary gradients in the static magnetic field along the scanner's three axes, and it is produced by three large coil systems, one for each axis. As the gradients are switched on and off the resulting gradient field varies with frequencies in the Hz to kHz range. The RF field consists of short pulses at certain intervals, and its carrier frequency is directly proportional to the strength of the static magnetic field, according to the proton gyromagnetic ratio of 42.58 MHz/Tesla. This mixture and intensity of magnetic fields is unique to the MRI environment and quite complex from a patient‐exposure perspective [Frankel et al., 2018].

There are well‐established acute effects of exposure to strong RF and gradient fields, such as tissue heating and neuroexcitation, and the current European safety guidelines for MRIs [CENELEC, 2016] are based on these immediate effects [Saunders et al., 1991; Reilly, 1992]. Specific absorption rate (SAR), measured in W/kg, is the parameter currently used to monitor and limit patient exposure to RF fields in the MRI environment, and it represents the average rate at which energy from the RF field is absorbed by the patient or tissue during any 6‐min period. The temperature increase in an MRI patient due to RF power deposition depends on individual body shape and composition. Current approaches to exposure assessment rely mainly on numerical methods for estimating the distribution of internal electric fields, currents, and SAR in the human body [Hartwig, 2015; Fiedler et al., 2018], but some experimental methods are also employed for SAR evaluation [Qian et al., 2013]. The rapidly switching gradient field induces electric fields in the body, which can cause uncomfortable peripheral nerve stimulation (PNS) and, at high levels, cardiac stimulation. The scientific grounds for possible long‐term effects from MRI exposure are lacking. Some in vitro studies have shown genotoxic effects while others have not been able to demonstrate any such results, leaving us without a clear conclusion about the state of MRI and genotoxicity [Fatahi et al., 2017]. While the interaction mechanisms for possible long‐term effects from magnetic field exposure are still unknown, epidemiological studies would help us understand if there are any long‐term effects. However, such research requires careful exposure characterization [Valberg, 1995], and when it comes to the complex exposure which occurs during an MRI exam, the concepts of exposure and dose need to be more clearly defined [Hansson Mild and Mattsson, 2017].

As a first step toward improved exposure assessment, we set out to characterize the MRI‐patient exposure environment in terms of exposure parameters that describe the RF and gradient magnetic fields involved. Our aim with this study was to clearly describe the exposure to the RF magnetic field and switched gradient magnetic fields in a set of MRI sequences, and show whether they could be stratified in terms of exposure level. In addition to inter‐sequence variation, we wanted to look at possible variation within a sequence, because the settings of the many sequence variables can be adjusted before starting a scan. If such adjustments have no impact on the resulting exposure, each sequence could be labeled with a fixed exposure value, which would be useful in epidemiological studies. However, if the variable settings modify exposure, MRI exposure assessment becomes more complicated and requires deeper analysis.

In the Swedish healthcare system, which serves a population of about 10 million, approximately half a million MRI exams are performed each year [Wilén et al., 2017], and an estimated 17,000 of those exams are performed on children aged 0–15 each year [Jorulf et al., 2015] (yearly estimate based on extrapolation from the number of MRI exams performed on children during a 2‐week period in 2011). Given that MRI is used increasingly in pediatric diagnostic imaging, a cohort study into the effects of MRI exposure on pediatric patients was recommended as a high‐priority goal in the latest SCENIHR opinion [SCENIHR, 2015]. In view of this, we chose to focus specifically on the exposure of children and adolescents, limiting the scope of this study to pediatric MRI protocols. We wish to illustrate the individual exposure characteristics of a group of sequences commonly used in pediatric MRI exams.

## BACKGROUND

### MRI Examination

RF and gradient fields are applied in different ways for different sequences, to accomplish various imaging goals such as high resolution, clear soft tissue contrast, and good signal‐to‐noise ratio (SNR). After a patient has been placed in the scanner and the MRI technician has entered the patient's information − including height and weight − into the scanner software, an examination protocol (including one or more MRI sequences) is chosen. For each sequence, the MRI technician selects the imaging slice(s) to cover the volume of interest. Field of view (FOV) is an adjustable sequence variable that determines how much of the patient will be included in the image. Other adjustable settings that determine the volume being imaged are slice thickness and slice spacing for 2D sequences, and locations per slab for 3D sequences. Adjustable timing variables include repetition time (TR), echo time (TE), and inversion time (TI), which determine how the gradients and RF pulses are spaced over time in the sequence. These settings affect the resulting image quality in terms of contrast and SNR. Additional adjustable variables include the receiver bandwidth (BW_r_), flip angle (FA), and number of excitations (NEX). The BW_r_ is the range of frequencies involved in the reception of the electromagnetic signal used to build the image. The FA indicates the amount of rotation of the scanned object's net magnetization when an RF pulse is applied, and a sequence can include multiple types of RF pulses, some with adjustable FA and some with fixed FA. NEX is the number of times data for an imaged slice are collected, and when slice data from a multi‐NEX sequence are averaged the SNR increases.

All of the adjustable settings mentioned here are central to the image production process, and, together with other sequence‐ and scanner‐specific variables, make up the unique characteristics of each MRI sequence. When all the adjustable scanner settings have been chosen by the MRI technician and the scan‐button has been clicked, a prescan is executed before the actual sequence. One purpose of the prescan is to calibrate the RF transmit gain, so it is optimized for the patient in the scanner. The scanner determines the right amount of RF power needed to precisely generate a known flip angle based on the patient's MR signal response which is directly related to the weight of the patient [McRobbie et al., 2007].

### Exposure

In the International Electrotechnical Commission (IEC) standard [CENELEC, 2016] three different scanner operating modes have been defined to regulate the intensity of the RF and gradient fields. Normal Operating Mode (NOM) is the most conservative scanner mode, and it allows a maximum whole‐body SAR (WB‐SAR) of 2 W/kg over any 6‐min period, which ensures that the patient should not experience heating. First Level Controlled Operating Mode (FLCOM) allows an SAR of 4 W/kg and can cause heating effects in some cases. Both NOM and FLCOM are used clinically, and NOM is chosen for especially sensitive patients, for example, small children and pregnant patients. Second Level Controlled Operating Mode (SLCOM) allows the highest level of exposure (>4 W/kg) and carries a significant risk of discomfort for the patient. Use of this mode requires explicit ethical approval and is only relevant for research studies, not for clinical use. SAR may not always be evenly distributed in the whole body, and higher local SAR values are allowed (averaged over any 10 g of tissue), such as 10 W/kg (head or trunk) or 20 W/kg (extremities) in NOM, and 20 W/kg (head or trunk) or 40 W/kg (extremities) in FLCOM, as long as the whole‐body SAR limits are also obeyed. The SAR value for a sequence is estimated by the scanner software based on the average power in the applied RF field (influenced by RF pulse design and sequence settings such as TR and FA) and the operator‐provided mass of the patient. This estimated SAR value is then compared to the operating mode‐specific SAR limit to ensure compliance with the regulations.

The likelihood of inducing PNS and cardiac stimulation is governed by the rate of change of the gradient field, dB/dt, and the effective stimulus duration, *t*_s,eff_, that is, the slope steepness and rise time of the increasing gradient field. The IEC safety limits for gradient fields regulate dB/dt to protect patients against cardiac stimulation in all three scanner operating modes. In FLCOM and NOM additional restrictions limit dB/dt to minimize the occurrence of uncomfortable PNS. In FLCOM the rate of change of the gradient field of a whole‐body gradient system is limited according to

(1) $$dB/dt=20\left(1+\frac{0.36}{t_{s,eff}}\right)\;T/s$$

where *t*_s,eff_ is the effective stimulus duration measured in milliseconds [CENELEC, 2016]. In NOM the dB/dt limit is reduced to 80% of that given in equation (1). These are important safety limits in their own right, but they tell us nothing about the dB/dt profile of a sequence, that is, whether dB/dt levels close to the limit occur frequently or rarely during a sequence.

SAR and dB/dt_max_ are the only two parameters commonly used to describe patient exposure in MRI today, and when such values are given for a sequence, they merely describe the upper limit for that particular sequence. If a sequence is said to have a WB‐SAR of 1.1 W/kg, it means that any 6‐min average of the sequence will be less than or equal to 1.1 W/kg. Hence we are not given the actual amount of RF exposure, and sometimes scanner‐reported SAR values are significantly overestimated compared to the actual amount of RF power delivered [El‐Sharkawy et al., 2012]. Similarly, the chosen scanner operating mode provides us with an upper limit for dB/dt, but not the actual maximum dB/dt value experienced during the sequence, only the maximum dB/dt value allowed for that sequence. SAR and dB/dt_max_ relate to direct effects of heating and neuroexcitation inside the body. In contrast, exposure parameters used in epidemiological studies for investigating long‐term effects often describe the EMF exposure environment outside the body, independent of patient size and shape [Röösli and Vienneau, 2014]. In this study, we chose a set of 14 such exposure parameters, which describe the magnetic fields outside the body, in an attempt to give a more comprehensive picture of the patient's exposure during an MRI procedure − without making any claims about which exposure parameters are the most biologically relevant. These parameters include mean, extreme, and cut‐off values, to describe the complex nature of the MRI exposure environment.

## MATERIALS AND METHODS

### Measurement Setup

**Figure 1:**
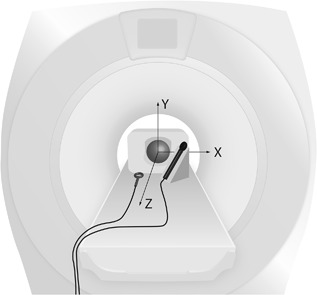
Measurement setup. The image shows the spherical phantom in the scanner isocenter, rectangular head coil around the phantom, gradient field‐measuring probe on a wooden stand to the right, and RF field probe on the table to the left. The three‐dimensional gradient field probe was placed at *x* = 13, *y* = 10, *z* = 32 cm relative to the scanner isocenter, and the one‐dimensional RF probe was placed at *x* = −10, *y* = −14, *z* = 34 cm relative to the scanner isocenter, facing the y‐direction since the RF field is perpendicular to the static field. The labeled arrows illustrate the directions of the scanner coordinate system.

RF and gradient fields were measured with pick‐up coils inside the bore of an integrated, 3‐Tesla SIGNA PET/MRI scanner (GE Healthcare, Chicago, IL) during seven different imaging sequences. The scanner was used solely for MRI in this study, without the PET function. The object scanned was a spherical, liquid‐filled phantom (model 2152220), with a diameter of 18 cm (General Electric Company, Milwaukee, WI). The phantom was placed in a 24‐channel receive‐only GE head and neck unit (GE Healthcare), and the measuring probes were placed just outside the head coil as shown in Figure 1, 36 cm (gradient probe) and 38 cm (RF probe) from the scanner isocenter, points where the gradient field would be non‐zero. Note that the chosen measuring points were not global maxima for magnetic field exposure, but simply positions in the space normally occupied by a patient during an MRI exam. The RF transmit body coil, which is integrated in the scanner, was used to produce the RF field. The maximum amplitude of the scanner's gradient coil system was 44 mT/m and the maximum slew rate was 200 T/m/s. All scans were run in NOM.

A Narda ELT B‐Field probe (IEC 62311 standard 3 cm^2^; Narda Safety Test Solutions, Pfullingen, Germany) with three orthogonal induction coils was used together with a handheld Narda ELT‐400 exposure level tester (Narda Safety Test Solutions) in B‐field measuring mode (settings: Mode 80 mT, Range Low, Low Cut 30 Hz) to measure the gradient field, shown as the probe to the right of the phantom in Figure 1. A one‐dimensional (5 cm diameter) induction coil of King design [Whiteside and King, 1964], built in‐house, was used to measure the pulsed RF field, shown as the probe to the left of the phantom in Figure 1. Long coaxial cables connected the measuring coils in the scanner room with the ELT‐400 and signal‐recording equipment in the adjacent operatoŕs room. Calibration was performed using the same cables in a transverse electromagnetic (TEM) cell in the appropriate frequency range.

All four signals (three gradient signals and one RF signal) were measured with a sampling frequency of 50 kHz by a Picoscope 5444B PC‐oscilloscope (Pico Technology, Cambridgeshire, United Kingdom) connected to a laptop computer running the oscilloscope software (Picoscope 6.11.12.1692). The resultant gradient B‐field was calculated according to

(2) $$B\left(t\right)=\sqrt{\left({B_{x}\left(t\right)}^{2}+{B_{y}\left(t\right)}^{2}+{B_{z}\left(t\right)}^{2}\right)}$$

where *B*_x_, *B*_y_, and *B*_z_ represent the individual signals from the orthogonal coils in the gradient‐field measuring probe.

### MRI Sequences

Seven different imaging sequences were chosen from brain scan protocols for children up to the age of 16. The protocols were provided by the radiology department of a major Swedish children's hospital and constituted the three most common brain scan protocols used at that clinic. The selected sequences include a wide range of sequence types (2D and 3D, spin echo and gradient echo, *T*_1_‐weighted and *T*_2_‐weighted, diffusion‐weighted, vascular, etc.), and they are listed in Table 1 with their default scanner settings and the range allowed for each setting.

**Table 1:** Default scanner settings^a^ for the seven sequences which were compared, with ranges for adjustable settings in parentheses^b^

Sequence ID	A	B	C	D	E	F	G
Scan time (min:sec)	3:13	2:16	3:46	4:03	8:12	6:24	2:23
Sequence name	T1 FLAIR	DWI	3D TOF	3D FIESTA	T1 SE	FSPGR BRAVO	SWAN
Sequence type	2D T_1_‐weighted fluid attenuation sequence with inversion recovery	2D Diffusion weighted sequence	3D Time‐of‐flight angiography sequence	3D Balanced steady‐state gradient echo sequence	2D T_1_‐weighted spin echo sequence	3D Ultrafast spoiled gradient echo sequence	3D Susceptibility enhanced angiography sequence
TR (ms)	2707.4	9096	11	5	6000	8.9	54.4
TE (ms)	24 (1–2000)	73.6 (73.6–207)	2.6 (0.1–11)	2.1	18 (18–20)	3.5	24.3 (22.7–25)
TI (ms)	827 (334–1244)	–	–	–	–	450 (30–450)	–
FA (°)	111 (1–147)	–	15 (1–45)	55 (1–90)	–	12 (1–25)	15 (1–50)
FOV frequency direction^c^ (cm)	22 (21–31.5)	24 (13.6–60)	22 (19.5–50)	18	22 (22–50)	26	20
FOV phase direction (cm)	19.8	21.6	16.5	16.2	19.8	26	20
Acquisition matrix (N_f_ x N_p_)	320 × 288	128 × 128	384 × 256	200 × 320	320 × 224	264 × 264	320 × 224
BW_r_ ^d^ (kHz)	41.67 (41.67–142.86)	250	62.5	62.5	31.25 (31.25–83.33)	31.25	62.5
Slice thickness (mm)	4 (3.8–10.4)	4.5 (4.5–20)	0.8 (0.6–5.2)	0.6	4 (1.1–10)	1 (0.84.6)	3 (0.8–3.1)
Slice spacing (mm)	0.5 (0.1–7.1)	0.4 (0.1–20)	–	–	0.5 (0.1–20)	–	–
Number of slices (2D) or locations per slab (3D)	32	32	104	98	30	244	48
NEX	2	–	–	1	2	1	–
WB‐SAR^e^ (W/kg)	1.11	0.1	0.07	1.05	0.82	0.07	0.06

^a^ Initial sequence settings for repetition time (TR), echo time (TE), inversion time (TI), flip angle (FA), field of view (FOV) in the frequency‐encode direction and in the phase‐encode direction, acquisition matrix size (N_f_ = number of pixels in the frequency encode direction, and N_p_ = number of pixels in the phase encode direction), receiver bandwidth (BW_r_), slice thickness, slice spacing, number of slices, and number of excitations (NEX) for sequences A‐G. Instances denoted with − indicate that the parameters were not adjustable or not part of those sequences.

^b^ The numbers in parentheses indicate the range allowed when changing one setting at a time without affecting any of the other settings or the total scan time. Default values without parenthesis indicate that a parameter was not variable in a given sequence, or that it was not possible to vary without affecting the total scan time.

^c^ FOV in the frequency‐encode direction was varied while keeping the acquisition matrix size (N_f_) constant which means that the pixel size varied.

^d^ Receiver bandwidth values represent the frequency range around the nominal radiofrequency reception center frequency, so BW_r_ = 250 kHz actually means ±250 kHz, that is, a frequency range of 500 kHz.

^e^ Whole‐body SAR (WB‐SAR) estimates were provided by the scanner before the start of the scan, and based on the fictitious data of a 35‐kg child instead of the 3‐liter phantom that was actually scanned.

### Data Acquisition

Imaging slices were selected in the transverse plane, along the *z*‐axis of the scanner, centered at the scanner's isocenter, and the frequency‐encode direction was set to anterior‐posterior (A/P), that is, aligned with the *y*‐axis of the scanner coordinate system. Other variable sequence settings, for example, FOV size, number of slices, and FA, were chosen based on the pre programmed sequence protocols we received from the radiology department, to simulate real‐life clinical MRI exams.

For inter‐sequence comparisons, all sequences were executed during equal conditions, with the same head coil, the same phantom, and the same placement of the measuring probes, to show variations between sequences at one specific point. Any maximum or minimum values acquired were specific to that point in space, and not necessarily maximum or minimum exposure values for the whole scanner. Each sequence was executed and measured three times.

To investigate the possible exposure variability within a sequence, we studied a few sequence variables individually: TE, TI, FA, Slice thickness, Slice spacing, BW_r_, and FOV. For each variable, the scanner setting was adjusted to cover the maximum span allowed by the protocol, while keeping all other settings constant, including total scan time. These exposure measurements were conducted in FLCOM for sequence A, at 4–6 different settings for each variable, and the fictitious patient height and weight entered were 160 cm and 60 kg.

### Data Handling and Analysis

The RF and gradient field signals were analyzed with the software Matlab, version R2016b (Mathworks, Natick, MA). As an average value of the entire sequence signal, the root mean square (RMS) was calculated for the RF field and gradient field. The maximum value was also registered, as well as the proportion of the gradient signal above an arbitrary cut‐off value of 8 mT to reveal differences in exposure.

RF pulses were located and documented, using the Matlab function “findpeaks” for pulse height and thresholding for pulse width. For each RF pulse in a sequence, the local pulse RMS value was calculated, and the widths of the RF pulses were used to calculate the duty cycle for the entire sequence in terms of RF pulse on/off.

**Figure 2:**
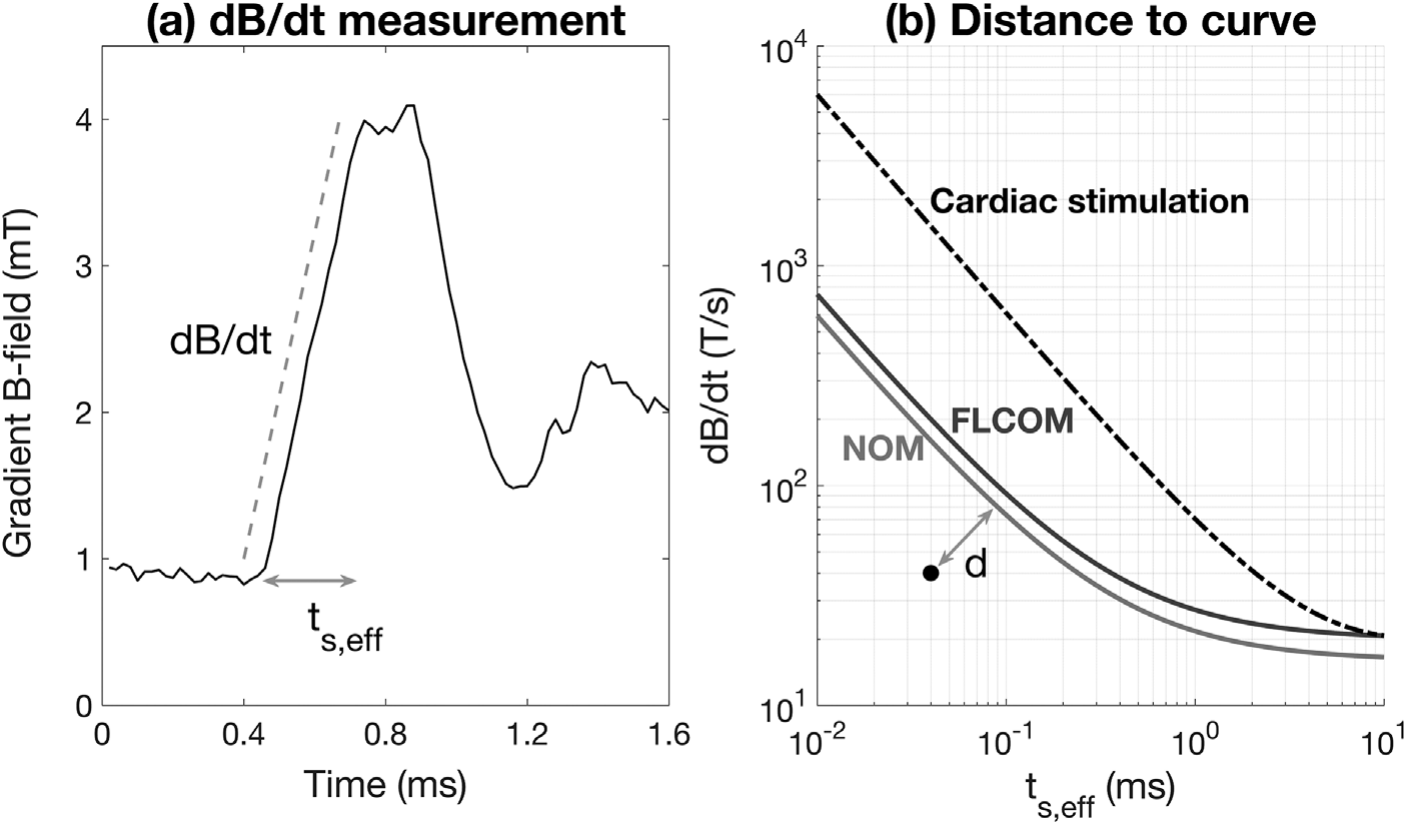
(a) shows how the time derivative of the gradient field, dB/dt, and the corresponding effective stimulus duration, *t*_s,eff_, were measured for each B‐signal peak. (b) shows the limits, in terms of dB/dt and *t*_s,eff_, applied to normal operating mode (bottom curve) and first‐level controlled operating mode (middle curve), and the limit for cardiac stimulation (top curve). The dot in the graph represents the rising or falling edge of one signal peak, and *d* is the minimum geometric distance from the dot to the normal operating mode‐curve.

Peaks in the gradient signal were located with the Matlab function “findpeaks,” and the steepness of the rising and falling edges of the signal peaks was measured to determine the rate of change of the gradient magnetic field, dB/dt, as illustrated in Figure 2. The duration of each rising or falling edge was measured to associate an effective stimulus duration (*t*_s,eff_) with each instance of measured dB/dt. The dB/dt was then plotted against the *t*_s,eff_ in a graph together with the NOM limit curve described in equation (1) so that the minimum distance (*d*) from each point to the curve could be calculated. The geometric distance *d* has no meaningful unit, but is an interesting exposure parameter as it takes into account both the dB/dt magnitude and the *t*_s,eff_. The proportion of the total signal with dB/dt values above a cut‐off of 15 T/s was also registered.

Despite analog low‐pass filters on the gradient field signal cables, the impact from sharp RF pulses was great, and it distorted the gradient field signal. Therefore, the measured gradient signal was replaced by an interpolated line whenever it coincided with an RF pulse, to omit distorted data from the analysis.

For each exposure parameter, the seven groups of repeated sequence measurements were compared using the non‐parametric Kruskal–Wallis test, to determine if the sequences differed significantly from each other.

## RESULTS

**Figure 3:**
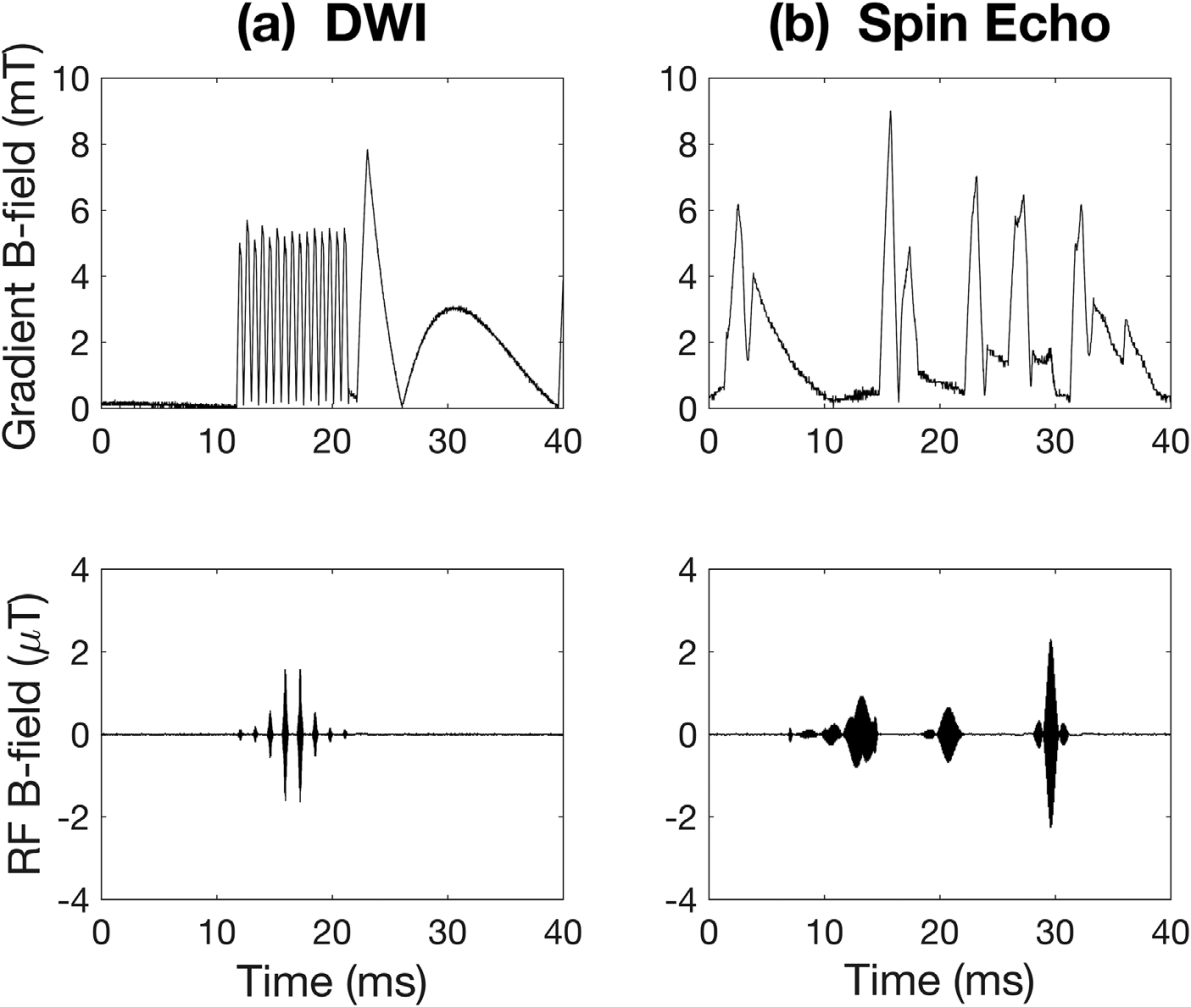
Two examples of sequence gradient field signals (top) and the corresponding RF signals (bottom). The signals shown are short segments from (a) a diffusion‐weighted imaging (DWI) sequence (sequence B in Table 1) and (b) a T_1_‐weighted spin echo sequence (sequence E in Table 1).

The gradient and RF fields of the seven sequences compared in this study exhibited different characteristics at close inspection of the measured signals. An example of this is shown in Figure 3 where 40‐ms segments from two different sequences are shown. Both the shape and “density” of the gradient field signals and the amplitude and spacing of the RF pulses varied markedly. Some sequences had an irregular composition, with long periods of no signal activity, as in the sequence on the left, while other sequences showed a regular pattern of evenly spaced peaks, as in the sequence on the right.

The exposure varied among the different sequences. Table 2 shows 14 exposure parameter results for each of the seven MRI sequences. The *p*‐values on the right indicate that, at a significance level of 0.05, there were differences between two or more sequences for all 14 exposure parameters. Sequence E, which was a *T*_1_‐weighted spin echo, was the sequence that most often exhibited the highest exposure level among the seven sequences, doing so for 5 out of the 14 exposure parameters. Sequence A, which was a 2D *T*_1_‐weighted fluid‐attenuated inversion recovery (FLAIR) sequence, did not rank highest on any exposure metric but did come in last for 4 of the 14 exposure parameters. Sequences B, C, D, F, and G each ranked highest on 1–3 parameters.

**Table 2:** Exposure parameter results^a^ for 7 sequences

Sequence ID	A	B	C	D	E	F	G	*p*‐value^b^
Gradient field exposure parameters								
RMS B (mT)	2.69 ± 0.02	3.83 ± 0.06	3.42 ± 0.01	4.96 ± 0.00	**7.85 ± 0.12**	2.93 ± 0.01	3.54 ± 0.02	0.003
Maximum B (mT)	15.8 ± 0.7	**36.9 ± 1.74**	9.72 ± 0.03	12.9 ± 0.51	36.0 ± 1.10	12.3 ± 0.74	9.73 ± 0.08	0.005
B >8 mT (%)	0.82 ± 0.02	4.54 ± 0.24	1.54 ± 0.02	7.41 ± 0.10	**19.9 ± 1.21**	1.68 ± 0.02	2.78 ± 0.04	0.003
Maximum slope dB/dt (T/s)	22.3 ± 0.20	28.9 ± 0.53	32.2 ± 1.56	**33.2 ± 1.10**	25.5 ± 0.14	24.0 ± 1.62	25.3 ± 0.92	0.006
Mean slope dB/dt (T/s)	13.5 ± 0.04	**17.2 ± 0.01**	14.5 ± 0.00	13.7 ± 0.05	11.3 ± 0.02	13.2 ± 0.00	13.0 ± 0.02	0.003
Minimum d to NOM curve	0.59 ± 0.01	0.24 ± 0.12	0.26 ± 0.00	0.24 ± 0.02	**0.04 ± 0.03**	0.38 ± 0.01	0.54 ± 0.05	0.006
dB/dt >15 T/s (%)	3.10 ± 0.02	6.49 ± 0.01	**9.56 ± 0.04**	3.04 ± 0.11	0.08 ± 0.00	2.59 ± 0.01	2.79 ± 0.06	0.004
Radiofrequency field exposure parameters								
RMS B1 (μT)	0.37 ± 0.00	0.13 ± 0.00	0.11 ± 0.00	**0.44 ± 0.00**	0.36 ± 0.00	0.11 ± 0.00	0.10 ± 0.00	0.003
Duty cycle RF (%)	23.0 ± 0.04	1.61 ± 0.00	6.78 ± 0.03	7.32 ± 0.02	**23.3 ± 0.04**	5.41 ± 0.04	0.74 ± 0.00	0.003
Maximum B1 pulse height (μT)	2.49 ± 0.02	3.44 ± 0.00	1.15 ± 0.00	3.68 ± 0.00	3.57 ± 0.02	1.85 ± 0.02	**4.75 ± 0.07**	0.003
Mean B1 pulse height (μT)	1.34 ± 0.01	1.12 ± 0.00	1.01 ± 0.00	3.35 ± 0.01	0.93 ± 0.01	0.99 ± 0.01	**3.53 ± 0.05**	0.003
Maximum B1 pulse width (ms)	8.62 ± 0.00	3.01 ± 0.01	2.83 ± 0.01	0.44 ± 0.00	4.83 ± 0.03	**16.0 ± 0.00**	0.64 ± 0.00	0.003
RF pulse frequency (per second)	137 ± 0.11	31.9 ± 0.05	95.1 ± 0.22	**202 ± 0.45**	181 ± 0.11	93.4 ± 0.03	18.6 ± 0.07	0.003
Maximum RMS within a pulse (μT)	1.51 ± 0.05	1.83 ± 0.06	0.39 ± 0.00	1.68 ± 0.00	**2.14 ± 0.02**	0.79 ± 0.01	1.22 ± 0.02	0.003

^a^ Mean ± standard deviation of gradient‐ and radiofrequency field exposure for three repeated measurements using the fixed set of adjustable settings for seven sequences. Bold numbers indicate which sequence ranked highest on exposure for each exposure parameter.

^b^ The *p*‐value gives the probability that there is no significant difference between the sequences according to the non‐parametric Kruskal–Wallis test.

The smallest difference in exposure among the sequences was found for the exposure parameter that described maximum gradient field slope dB/dt, where sequence D (which ranked highest) had a 50% higher value than sequence A (which ranked lowest). The largest difference was exhibited by the RF duty cycle exposure parameter; when total scan time (found in Table 1) was taken into account, sequence E produced a 107‐fold longer total RF field exposure than sequence G. When comparing the number of pulses, sequence E had 33 times more pulses than sequence G.

Summarizing the exposure of a full MRI exam containing the seven sequences in Table 1, measured at the position specified in Figure 1, resulted in a total of 2.3 min with gradient field levels above 8 mT, 58 s of dB/dt rates above 15 T/s, a maximum dB/dt of 33.2 T/s, and a total of 2.29 × 10^5^ RF pulses spanning a total of 3.5 min of RF field exposure.

**Figure 4:**
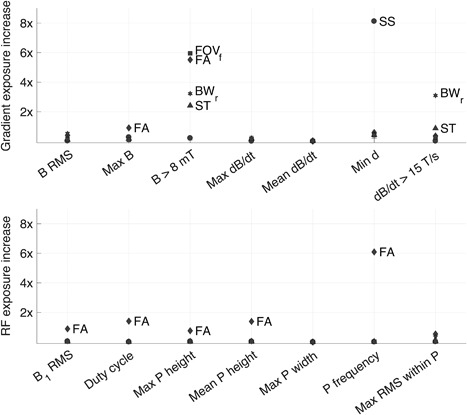
Impact of adjustments to sequence settings on gradient field exposure parameters (top panel) and RF exposure parameters (bottom panel) for sequence A. Each marker shows how many times the exposure increased resulting from adjustments to one particular setting, and the exposure increase was calculated as (highest exposure value − lowest exposure value) / lowest exposure value. Sequence settings varied were echo time (TE), inversion time (TI), flip angle (FA), slice thickness (ST), slice spacing (SS), receiver bandwidth (BW_r_), and field of view in the frequency‐encode direction (FOV_f_), according to the ranges noted in Table 1. Only markers representing an exposure increase of >70% are labeled. Exposure parameters are listed (from left to right) in the same order as in Table 2, and P stands for pulse in the RF exposure parameter labels.

The within‐sequence investigation also indicated some exposure variation. Figure 4 shows how adjustments to certain sequence settings resulted in 6–8‐fold increases in exposure during sequence A, while other settings had no impact on exposure. For example, the proportion of gradient field signal above 8 mT increased 5.5 times with a change in FA and six times with a change in FOV. The minimum distance (*d*) to the NOM curve changed by a factor of eight for different slice spacing settings. These are examples of within‐sequence exposure variability that resulted when the scanner settings were changed.

**Figure 5:**
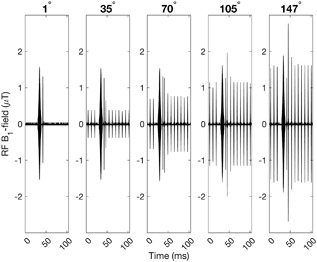
100 ms RF‐signal segments from sequence A performed with different flip angles. The amplitude of the train of narrow pulses increases with increasing flip angle.

Figure 5 shows a section of an RF signal with five different flip angle settings ranging from FA = 1° to FA = 147° and Table 3 gives some exposure measurements from when the flip angle was varied. These numbers provide a closer look at some of the flip angle‐data presented in Figure 4 by showing how the exposure was affected by flip angle variation. The six‐fold difference in RF pulse frequency noted in Table 3 is illustrated by Figure 5, which shows how the amplitude of the pulse train decreased with decreasing flip angle and disappeared completely at the lowest FA setting.

**Table 3:** Exposure parameter results for different flip angles in sequence A^a^

	Flip angles						
Exposure parameters	1°	35°	70°	105°	111°	147°	Exposure increase (%)
B >8 mT (%)	0.39	0.38	0.38	0.45	0.60	2.50	558
Minimum d to NOM curve	0.49	0.50	0.37	0.40	0.36	0.32	57
dB/dt >15 T/s (%)	2.87	2.86	2.86	3.04	3.12	3.88	36
RF duty cycle (%)	9.75	17.8	20.7	22.2	22.5	23.5	141
Mean RF pulse height (uT)	1.37	0.57	0.89	1.01	1.06	1.22	139
RF pulse frequency (pulses per second)	24.1	130	127	156	157	171	609
WB‐SAR estimate^b^	0.60	0.65	0.80	1.05	1.11	1.49	148

^a^ Gradient and RF exposure for sequence A performed with different flip angle settings. The exposure increase for each exposure parameter was calculated as 100 * (highest exposure value − lowest exposure value) / lowest exposure value.

^b^ Whole body SAR (WB‐SAR) as estimated by the scanner based on sequence settings and an operator‐entered fictitious patient weight of 60 kg.

Table 3 includes whole‐body SAR estimates for each of the six FA settings, increasing 148% over the full range, which is similar to the impact of flip angle variation on RF duty cycle and mean RF pulse height. TI was the only other adjustable scanner setting that affected the SAR estimate, and in that case, SAR only decreased from 1.11 to 1.07 W/kg with increasing TI.

## DISCUSSION

The sequences compared in this study differed from each other with respect to every exposure parameter that we measured. These differences, which were significant between at least two sequences for every exposure parameter, ranged from 50 to 10,000% between the highest and lowest ranking sequences, and can be found among the sequences in a single MRI exam protocol. In reality, a protocol could include all seven sequences or more, or it could consist of only one or two. The choice of which sequences to include can substantially affect the total amount of exposure during an MRI exam.

The exposure parameters express different aspects of the RF and gradient field exposure, as shown by the fact that six out of the seven sequences took turns ranking highest on different exposure metrics (bold numbers in Table 2). Stratification of the sequences by exposure level, therefore, depends on which parameter is used to represent the exposure.

These variations show that sequences can be quite different from each other in terms of exposure, and here we have only compared a small group of sequences on one type of scanner. There are many other types of sequences that did not fit into this study, and there are several different types of scanners on the market, so the differences between MRI sequences may be even more pronounced in a larger sample.

Variation in sequence settings (listed in parentheses in Table 1) can have a pronounced impact on exposure, as was illustrated by the 5–8 fold exposure increase caused by variations in flip angle, slice spacing, and FOV. The within‐sequence exposure differences documented for sequence A, which could be as large as 60% of the corresponding between‐sequence differences, are not negligible and must be taken into account when conducting exposure assessments of the MRI environment. This means that the same sequence can cause different amounts of exposure depending on the initial settings chosen by the MRI technician.

There are many different adjustable settings in an MRI sequence, some of which are listed in Table 1 together with their default values for sequences A–G. Because the settings can be varied, and sometimes widely, there is definitely a potential for intra‐sequence exposure variability. However, the full range may not always be clinically relevant. In sequence A the flip angle had a very wide adjustable range, from FA = 1° to 147°, but such a wide range is not usually used clinically. The default value was 111°, and changing the flip‐angle setting too much could affect image quality negatively. Therefore, in some cases, the wide range in the adjustable settings found in Table 1 may only be of academic interest. Even though FA adjustments are sometimes made to lower the SAR of a sequence, it may be argued that the large difference in RF exposure exhibited when turning the flip angle down from 111° to 1°, which reduced the RF pulse amplitude to background noise level, is irrelevant to exposure assessment because such a big adjustment would never be made clinically. However, the fact that scanner settings can be adjusted (exemplified in Table 1), and that such adjustments can impact exposure (shown in Fig. 4), tells us that the total exposure from a sequence is not determined until all the scanner settings have been chosen, which makes MRI exposure specific to each exam occasion. Furthermore, because the RF power is adjusted to fit the size of the patient during the prescan, a sequence may provide different levels of exposure to a large adult and small child even if the sequence settings are identical − so MRI exposure is also patient‐specific. A study sample with more than one sequence might have given us further examples of within‐sequence variation with clearer connections to clinical practice, but the small example shown in this study is enough to illustrate the important point that a meaningful exposure assessment of MRI requires more information than merely the sequence type.

The settings that were varied in this study of within–sequence exposure variability are a subset of all the settings that can be adjusted before a scan. Some settings cannot be adjusted without directly impacting other variables, but such examples were not included in our analysis to limit the scope of the project. This interconnectivity among sequence variables introduces yet another layer of complexity into the process of exposure assessment and makes it difficult to predict exactly how an adjustment to the settings will affect the total exposure level. Even though standard pre programmed settings exist, adaptation of a sequence to the individual patient is done regularly, to varying degrees, depending on clinic tradition and availability of MRI‐application expertise. For example, different scanning modes are chosen depending on the patient. The FOV is adjusted to fit the body region of interest, often with a preserved resolution which requires corresponding adjustments to the size of the acquisition matrix. NEX can be increased to improve the signal‐to‐noise ratio, and many other adjustments are made regularly. Sensitive patients may require more advanced adjustments to a sequence, such as SAR‐reducing adjustments to the RF field, to prevent heating of certain kinds of implants or fever in patients who have a reduced ability to regulate body heat. For patients with sensitive hearing, a sequence can be run in noise‐reducing mode, which, in addition to reducing the noise level, also reduces the time derivative of the gradient magnetic field [Wilén et al., 2010].

All of these examples from day‐to‐day clinical practice are mentioned to support our claim that MRI exposure is not only sequence‐specific but also specific to each patient and exam occasion. Although the concept of patient exposure in the MRI environment is quite complex, the exposure can be described with parameters that differentiate between varying levels of gradient field exposure and between varying types of RF exposure. The exposure parameters listed in Table 2 were chosen to demonstrate the full exposure of a sequence, using a combination of mean, extreme, and cut‐off values.

The SAR values in Table 3 increased linearly with the flip angle. This was expected as the SAR estimate depends on the average RF field power, which in turn depends on the shape and spacing of the RF field pulses, while the shape of an RF pulse (including amplitude and duration) is what determines the flip angle resulting from such a pulse. Duty cycle and mean pulse height were other RF exposure parameters that varied with flip angle to a similar extent, but not quite as linearly due to the disappearance of the adjusted pulse into background noise at FA = 1°. Interestingly, a gradient field exposure parameter (proportion of B >8 mT) increased substantially with FA, especially between 111° and 147°, hinting at the complex interaction between the different components of an MRI sequence [Bernstein et al., 2004].

SAR‐ and dB/dt_max_ values provide important information about a sequence, ensuring compliance with current safety regulations. However, because such values are only upper limits, they do not provide a description of the actual exposure in a sequence. A measured dB/dt value that is close to the dB/dt_max_ limit may be an unusual one‐time occurrence, or it may be repeated frequently during the length of a sequence. Likewise, the given SAR value of a sequence only shows an estimate of the average energy deposition rate but discloses nothing about the way in which the energy was delivered − in small but frequent pulses or one big burst. For meaningful exposure assessment, relevant to future epidemiological research, exposure metrics that describe the complete sequence are needed. Perhaps some of our suggested exposure parameters can be useful.

The small number of sequences included in this study could be considered a limitation, and they were only measured in normal operating mode and only on a single scanner. These choices were based on our intention to study a typical clinical situation of pediatric patient exposure. A larger selection of sequences, and measurements in first‐level controlled operating mode, could have resulted in even larger inter‐sequence differences and higher exposure values. It should also be noted that even though the sequences in this study were chosen from a pediatric scan protocol, they are not used exclusively for pediatric patients. Thanks to the many adjustment possibilities, most sequences can be individually modified to suit almost any patient.

Another limitation of this study was the scope of the investigation of within–sequence variation, where the impact on exposure from varying initial sequence settings was only presented for one sequence. Within–sequence variation measurements from multiple sequences would have been interesting − to see if sequence settings affect exposure differently in different sequences. However, the results from a single sequence (Fig. 4) were adequate to show that varying the sequence settings can impact exposure.

A measurement limitation that resulted from the choice of lower frequency range limit (LOW CUT) at 30 Hz was the sometimes wavy appearance of the gradient field signal seen at the end of the signal segment in Figure 3a. This rounded shape deviates from what would typically be expected from an applied gradient, and because these signal segments might have a limited impact on exposure parameter results, it could be worth trying lower frequency range limits in future studies. The 30 Hz LOW CUT was originally chosen to avoid unnecessary noise but prevented the measurement equipment from interpreting lower (<30 Hz) frequencies properly, so perhaps a limit of 10 Hz or even 1 Hz may work better for future measurements. Another notable result was the high maximum gradient B‐field values of sequences B and E (see Table 2) when considering the maximum gradient performance of the scanner, 44 mT/m. If gradients of 44 mT/m were applied in all three directions simultaneously, one might expect the resultant gradient B‐field to be approximately 16 mT in the measurement position given in Figure 1, according to equation (2) with *B*_x_ calculated as 44 mT/m * 0.13 m, etc. However, we measured resultant gradient B‐fields of over 36 mT in that position. An explanation for this could possibly be found in the concomitant fields that necessarily occur when a gradient is activated [Hidalgo‐Tobon, 2010]. According to Maxwell's equations, the application of a gradient field in one direction will always be accompanied by transverse components that contribute to the total magnetic field. These additional fields are undesirable from an imaging perspective and may in some cases require compensatory action [Yablonskiy et al., 2005; Tao et al., 2017]. From an exposure perspective, concomitant fields must always be considered as they contribute to the total exposure of the patient.

It is not possible to determine, from the results of this study, which exposure metric would be the most relevant in an epidemiological research study. Nor is it within the scope of this study to determine what inter‐sequence difference in exposure level should be considered as biologically relevant. We are merely attempting to show that a set of commonly used MRI sequences can be stratified according to their level of exposure, and we are suggesting a few exposure parameters that might be useful in future epidemiological studies, as they give some insight into the characteristics of the full sequence. Our findings may also be useful in the design of cell‐exposure studies where careful exposure assessment is critical. A major criticism of past studies on MRI and genotoxicity has been the lack of adequate exposure characterization [Foster et al., 2017], and our results shed some light on the level of detail needed to fully describe patient exposure during an MRI exam.

Individualized exposure assessments through manual measurement for each combination of sequences and sequence settings would be prohibitively expensive and time‐consuming, and therefore not feasible on a large scale. However, since MRI exposure varies within, as well as between, sequences, exposure assessment needs to be performed on a patient‐ and exam occurrence‐specific basis. Information about the RF and gradient magnetic fields is processed and stored by the scanner during an MRI exam, and that information is used for SAR‐ and dB/dt calculations to ensure compliance with safety limits at all times. If such information were extracted and linked to useful exposure parameters, it may be possible to develop methods to determine the total exposure from a sequence, given the specific adjustable settings used for an individual patient. It would be worth investigating the possibility of automating such MRI exposure assessment for the benefit of future epidemiological research.

## CONCLUSION

Significant differences in exposure exist between MRI sequences. There is also within‐sequence variability, so it does not make sense to simply classify MRI exposure on a sequence level. Hence, MRI patient exposure is not only sequence‐specific but is also specific to the individual patient and exam occurrence, and this complexity requires careful consideration for an MRI exposure assessment to be meaningful.

Manual exposure assessments of every possible combination of MRI sequence settings would be prohibitively expensive and time‐consuming, and further studies will reveal if there are useful methods for automating the process of extracting useful information from a scanner during an MRI exam, for use in a meaningful exposure assessment relevant to epidemiological research.
